# Single-blind, randomized study comparing clinical equivalence of trulene and prolene polypropylene sutures in elective primary coronary artery bypass graft surgery

**DOI:** 10.1186/s13019-022-02095-z

**Published:** 2022-12-16

**Authors:** Ravi Shankar Shetty, Ajay Kaul, Aayush Goyal, Govardhan Reddy Konda, Sushant Srivastava, Ashok Kumar Moharana, T. S. Deepak

**Affiliations:** 1 Department of Cardio-Thoracic and Vascular Surgery, M S Ramaiah Medical College and Hospitals, Bangalore, Karnataka 560054 India; 2 Department of Cardio-Thoracic and Vascular Surgery, BLK-Max Super Speciality Hospital, New Delhi, 110005 India; 3 Clinical Affairs, Healthium Medtech Limited, Bangalore, Karnataka 560064 India

**Keywords:** Coronary anastomosis, Coronary artery bypass graft surgery, Coronary artery disease, Major adverse cardiac and cerebrovascular events, Polypropylene suture

## Abstract

**Background:**

Coronary artery bypass graft surgery (CABG) is one of the principle therapies for coronary artery disease, as it improves survival rate and quality of life (QoL). Polypropylene suture is commonly used in vascular and cardiac surgeries for anastomosis due to its long-term tensile strength and minimal tissue trauma. This study compared the clinical equivalence of Trulene® (Healthium Medtech Limited) and Prolene® (Ethicon-Johnson & Johnson) polypropylene sutures regarding incidence of myocardial infarction, stroke, renal failure and cardiac death (MACCE) occurring up to 26 weeks’ period post-CABG surgery.

**Methods:**

This multicenter, prospective, two-arm, parallel-group, randomized (1:1), single-blind study (n = 89) was conducted between August 2020 and September 2021. The primary endpoint, post-surgery cumulative incidence of MACCE was evaluated. In addition, anastomotic revision, surgical site infection (SSI), operative time, length of post-operative hospital stay, repeat revascularization, intraoperative suture handling characteristics, time taken to return to work and resume normal day to day activities, subject satisfaction score and QoL, and other adverse events were also recorded.

**Results:**

A total of 80 (89.89%) males and 9 (10.11%) females participated in the study. No incidence of MACCE was recorded in any of the study participants. Non-significant difference was observed in anastomotic revision, SSI, operative time, post-operative hospital stay, revascularization, return to work and normal day-to-day activities, subject satisfaction score and QoL, and intraoperative handling parameters (except ease of passage) between the treatment groups, Trulene® and Prolene®. Compared to screening visit, proportion of subjects with ‘no problems’ for each QoL dimension and the mean visual analogue scale increased with each subsequent follow-up visit.

**Conclusion:**

Trulene® polypropylene suture is clinically equivalent to Prolene® polypropylene suture and is safe and effective for anastomosis construction in CABG surgery during a routine clinical procedure.

*Trial registration* CTRI Registration No.: CTRI/2020/05/025157 (Registered on: 13/05/2020).

## Background

Over the last 25 years, India has witnessed 34% higher mortality rate in patients with cardiovascular and ischemic heart disease [1]. Coronary artery bypass graft (CABG) surgery, one of the most common elective surgical procedures, is considered to be safe in individuals with coronary artery disease (CAD) [2, 3]. CABG offers survival to patients with CAD, but the surgery itself is associated with post-operative complications, morbidity and mortality [4]. Myocardial infarction, stroke, graft stenosis, renal failure, repeat revascularization and death are the most commonly occurred post-CABG complications, and defined as major adverse cardiac and cerebrovascular events or MACCE [2]. Cardiac-death was reported in 2.2% [5], 13.31% (high risk patients) [6], and 3.2% [7] patients within 30 days of CABG surgery. According to Society of Thoracic Surgeons adult cardiac surgery database, 1.3% incidence of stroke was observed after CABG [8]. The Swedish Register of Information and Knowledge about Swedish Heart Intensive Care Admissions study showed 3.7% incidence of ischemic stroke 90 days post-CABG surgery [9]. Once the risks of myocardial infarction, stroke and renal failure have been assessed, this surgical procedure appears to be a good choice for improving the quality of life (QoL) of people with CAD [10]. A long-term follow-up study documented durable and positive effects of CABG surgery on patient’s physical and mental components of QoL [11].

The traditional on-pump or the newer off-pump techniques are usually opted for CABG surgeries; both involves blood vessel harvesting from the chest, arm or leg [12]. A registry-based study found similar short term (30 days) and mid-term (3 years) mortality rate along with incidence of MACCE in patients who underwent isolated CABG using either of the techniques. However, the cost-effective off-pump CABG was reported to be associated with shorter duration of hospital stays, lesser requirement for intensive care unit (ICU) admission and reduction in intubation time [13]. Median sternotomy is the gold standard approach for cardiac surgical procedures in children and adults, but without the cosmetic benefit that is achieved only by minimally invasive cardiothoracic surgery as in thoracotomy [14]. Though the practice of minimally invasive CABG is increasing, but median sternotomy is primarily performed because of its technical ease in delicate vascular dissection and suturing, exposing multiple areas of the heart, arteries and aorta [15]. In median sternotomy, left internal mammary artery is used as a conduit, along with other grafts viz. saphenous vein out of the lower extremities, right internal mammary artery, the radial artery, and the gastroepiploic artery depending on the location of the arteries. Radial artery graft and left internal mammary artery has longer patency (> 90%) [16]. Polypropylene monofilament is commonly used to anchor the graft at each end of the coronary arteriotomy that increases the patency of the proximal and distal coronary arterial lumens [17]. Polypropylene suture is stronger than nylon and offers better overall wound security [18–20]. An international, multi-centre, prospective cohort study by Ursulescu evaluated the performance of a polypropylene suture Optilene for anastomosis construction in CABG surgery with respect to the incidence of MACCE [2]. However, no randomized study has compared two commonly used brands of nonabsorbable polypropylene sutures for anastomoses repair following CABG surgery in Indian population.

The present study is designed to compare the clinical equivalence of Trulene® (Healthium Medtech Limited) and Prolene® (Ethicon-Johnson & Johnson) polypropylene sutures for anastomosis construction in CABG surgery using a routine clinical procedure till 26 weeks post-surgery. Both the sutures are sterile, monofilament, non-adhering, non-absorbable, synthetic in nature, and prepared from synthetic linear polyolefin. They are indicated for soft tissue approximation like in hernia repair, permanent fixtures, general surgery, microsurgery, orthopedic surgery, cardiovascular surgery, plastic surgery, ophthalmic and neurological procedures. Safety and efficacy parameters commonly used in CABG surgery were employed to evaluate the performance of the suture material.

## Methods

### Study design

The study was designed as a multicenter, prospective, two-arm, parallel-group, randomized (1:1), single-blind study. The study was conducted between 21^st^ August 2020 and 18^th^ September 2021. The primary objective was assessment of the performance of Trulene® and Prolene® polypropylene sutures with respect to cumulative incidence of MACCE till 26 weeks post-surgery. Secondary objectives included incidence of individual components of MACCE, surgical site infection (SSI), frequency of repeat revascularization (due to suture line bleeding), overall intraoperative handling, overall subject satisfaction score and QoL, tissue reaction, and other adverse events among the two groups.

### Ethical approval

In accordance with the Declaration of Helsinki, this study was registered with Clinical Trial Registry of India (CTRI Registration No. CTRI/2020/05/025157; Registered on: 13/05/2020). A clinical study protocol was developed a priori but not published in a peer-reviewed journal. The final study protocol was approved by the Ethics Committees of the participating sites. The study was designed, conducted, recorded, and reported in accordance to guidelines of ICH-GCP E6 R2, EN ISO 14155:2020, Indian MDR rules 2017, MDR (EU) 2017/745, New Drugs and CT rules 2019, and Consolidated Standards of Reporting Trials (CONSORT).

### Study participants

Male and female subjects, aged above 18 years, and scheduled for elective primary CABG surgery were included after obtaining written informed consent.

Subjects with a history of CABG, requiring other combined cardiac procedure, having an active infection at or around the skin incision site, with a history of allergy to polypropylene or similar products, with bleeding disorders, mental disorder, learning disability, or language barrier, and those who already participating in another cardiovascular study were excluded from the study. Furthermore, subjects who have received an experimental drug or used an experimental medical device within 30 days prior to the planned start of procedure, or subjects, who were unlikely to comply with the surgical procedure or complete the scheduled follow up visits, according to the investigator were excluded from the study. A total of 92 subjects, requiring elective primary CABG surgery were enrolled at two different centers.

### Study settings

The study was conducted at two sites: (i) Department of Cardio-Thoracic & Vascular Surgery, MS Ramaiah Medical College and Hospitals, Bengaluru, Karnataka, India, and (ii) Department of Cardio-Vascular Thoracic Surgery, BLK-Max Super Speciality Hospital, New Delhi, India.

### Intervention

Both Trulene® suture (Healthium Medtech Limited) and Prolene® suture (Ethicon-Johnson and Johnson) are sterile, monofilament, non-adhering and biologically inert polypropylene surgical sutures. Both the sutures are suitable for use in general soft tissue approximation.

### Study procedure

CABG surgery was performed either on-pump or off-pump. All routine aseptic precautions according to the existing standards, were followed preoperatively, perioperatively and post-operatively. Either midline sternotomy or a minimally invasive approach was used. Coronary arteries with clinically significant proximal stenoses and patent distal vessels were considered potentially suitable for grafting. Arterial and/or venous graft was used to redirect blood to an area of the coronary artery, distal to the blockage. The choice of graft was based on the surgeon’s preference, experience, and morphological characteristics of the graft, as well as on the coronary arteries and subject’s age and co-morbid conditions. During the procedure, each epicardial coronary artery containing a proximal stenosis was evaluated by direct external inspection and palpation for a suitable distal target site. An incision was then made in the coronary artery distal to the stenosis, and the bypass graft was anastomosed end-to-side to the incision. Both the distal and proximal anastomoses were sutured with either Trulene® or Prolene® polypropylene suture material using continuous suture technique. Drain was placed in all subjects during the surgery. Total seven visits including one screening visit and six study visits were conducted for assessing the outcomes. Subjects were examined on baseline visit (Day 0 or Day of surgery), Day 3, Day 4–15 (Day of Discharge or DOD), Week 6 post-DOD, Week 12 post-DOD and Week 24 post-DOD.

### Baseline demographics and other relevant characteristics

Age, gender, ethnicity, weight, height, vital signs, history of alcohol and tobacco use, previous percutaneous coronary intervention (PCI), and family history of cardio-vascular disease were recorded. American Society of Anaesthesiologists (ASA) classification and New York Heart Association (NYHA) grading, along with reason of the surgery were registered for both groups. Medical and surgical histories were noted, as well as physical examination (regarding central nervous system, respiratory system, gastrointestinal system, skin, joints and extremities, ear, nose and throat, general appearance, edema and lymph nodes) was done.

### Study outcomes

#### Primary endpoints

The primary outcome was composite evaluation of all MACCE including myocardial infarction, stroke, renal failure and cardiac death. This was recorded in the hospital up to the day of discharge and on all post-discharge visits till 26 weeks post-surgery. In case any event of myocardial infarction, stroke and renal failure resulted in cardiac death, then it was recorded under death rate. In the context of primary endpoint components, clinical acumen based on history and examination were used to assess MACCE.

#### Secondary endpoints

Frequency of repeat revascularization and anastomotic revision (refers to any CABG and/or PCI performed for any graft and/or stent-related complication as well as for progression of CAD) due to anastomotic bleeding, false aneurysms or other causes, along with incidence of SSI (either superficial or deep infection or organ-space infection according to Centers for Disease Control and Prevention criteria) was recorded at all post-operative visits. Operative time (length of surgery) as well as length of post-operative hospital stay (day of surgery to DOD) were noted. Intraoperative handling characteristics of the suture viz. assessment of ease of passage through tissue, first-throw knot holding, knot tie-down smoothness, knot security, stretch capacity, memory, suture fraying and tissue drag of both sutures were rated by the Investigator on a five-point scale as follows: 1 poor; 2 fair; 3 good; 4 very good; and 5 excellent. Time taken to return to work, and normal day to day activities was determined by questioning the subjects. Moreover, subject satisfaction score and QoL were recorded by questioning the subjects according to the EQ-5D-3L instrument (part 1), at screening visit and all post-discharge follow-up visits. This system comprises five dimensions: mobility, self-care, usual activities, pain/discomfort and anxiety/depression, which was indicated by the subject himself/herself. The EuroQoL-visual analogue scales (EQ-VAS) takes values between 100 (best imaginable health) and 0 (worst imaginable health), on which the subjects provided a global assessment of their health. Subject-reported condition/quality was measured using EQ-VAS, part of EQ-5D.

In addition, other standard details about intervention approach, type of operation and graft, number of grafts per subject, number of anastomoses, suture size, number of sutures used for each size, intra-aortic balloon pump (IABP) usage, number of blood transfusions per subject, perioperative complications (bleeding and others) and suture-related challenges were also recorded. Any untoward medical occurrence, unintended disease or injury, or untoward clinical signs (including abnormal laboratory findings) in subjects were collected as adverse event only if they were not reported as study endpoints. Adverse events that led to death or serious deterioration in the health was recorded as serious adverse events.

### Sample size

Cumulative proportion of complications of 9.8% till three months was reported [2], which was considered for the Prolene® suture arm. The anticipated cumulative proportion of the complications in the Trulene® suture arm till three months was assumed as 10.0%. With Type I error as 5%, 80% power and 5% margin of non-inferiority, the sample size requirement was calculated as 39 in each arm giving a total sample size requirement as 78. Further, keeping in view, drop out and post-randomization exclusion of 20% the required sample size was increased to 94 with 47 subjects to be enrolled in each arm. In order to adjust the sample to even number for block randomization, 46 subjects were enrolled in each arm. So, a total of 92 subjects participated in this study.

Sample size calculation formula:

$${\text{Two-sample}}\,{\text{parallel}}\quad {\text{Non-inferiority}}\quad {\pi }_{1} - {\pi }_{2} \ge {\delta }\quad {\text{n}}_{{\text{i}}} = \frac{{\left( {{\text{z}}_{{\alpha }} + z_{{\beta }} } \right)^{2} \left( {{\pi }_{1} \left( {1 - {\pi }_{2} } \right) + {\pi }_{2} \left( {1 - {\pi }_{2} } \right)} \right)}}{{\left( {{\pi }_{1} - {\pi }_{2} - {\delta }} \right)^{2} }}$$

n_i_: sample size required in each group, Z_α_: conventional multiplier for alpha, Z_β_: conventional multiplier for power, π_1_: cumulative incidence of MACCE in the standard Prolene® arm, π_2_: cumulative incidence of MACCE in Trulene® arm, δ: Margin of non-inferiority difference, π_1_–π_2_: size of difference of clinical importance.

### Randomization and blinding

Two independent random lists were generated (n = 46) (23 vs. 23) by a freely available random allocation software, using block sizes of 4, 6 or 8. All sequences were checked for correctness, and the lists were randomly assigned to the centers as required. The subjects were randomized using block randomization to ensure an unbiased treatment assignment in a 1:1 ratio to either Trulene® (n = 46) or Prolene® (n = 46) suture group. Before the initiation of the study, an interactive, automated randomization number was generated by sequentially numbered opaque sealed envelopes, and the randomization codes were issued to the sites in sealed envelopes.

This was a single-blind study and subjects were kept blind to the allocation status. The operating staff cannot be blinded to allocation because of the nature of the intervention. But they were instructed not to disclose the allocation status of the participant at any time.

### Statistical analysis

The subjects were analyzed using the per-protocol or PP principle, which consist of all subjects who had complete data on the primary effectiveness parameter at 26 weeks’ follow-up and no major protocol deviations (i.e., having an impact on the primary endpoint). All continuous variables were expressed as mean ± SD and compared using the t-test for normally distributed data, and Mann–Whitney U test for distribution-free data. All qualitative variables were expressed as proportions or percentages and were compared using Chi-square test or Fisher's Exact test. A p-value of < 0.05 was considered statistically significant. The primary endpoint, incidence of MACCE was summarized as proportion/percentage and was compared using Fisher's Exact test. Secondary endpoints were expressed as mean ± SD or as proportions/percentages based on quantitative or qualitative nature of variable. The additional subgroup analysis was done using Chi-square test. All analyses were carried out with SPSS version 28.0 (SPSS, Chicago, Illinois, USA).

## Results

**Fig. 1:**
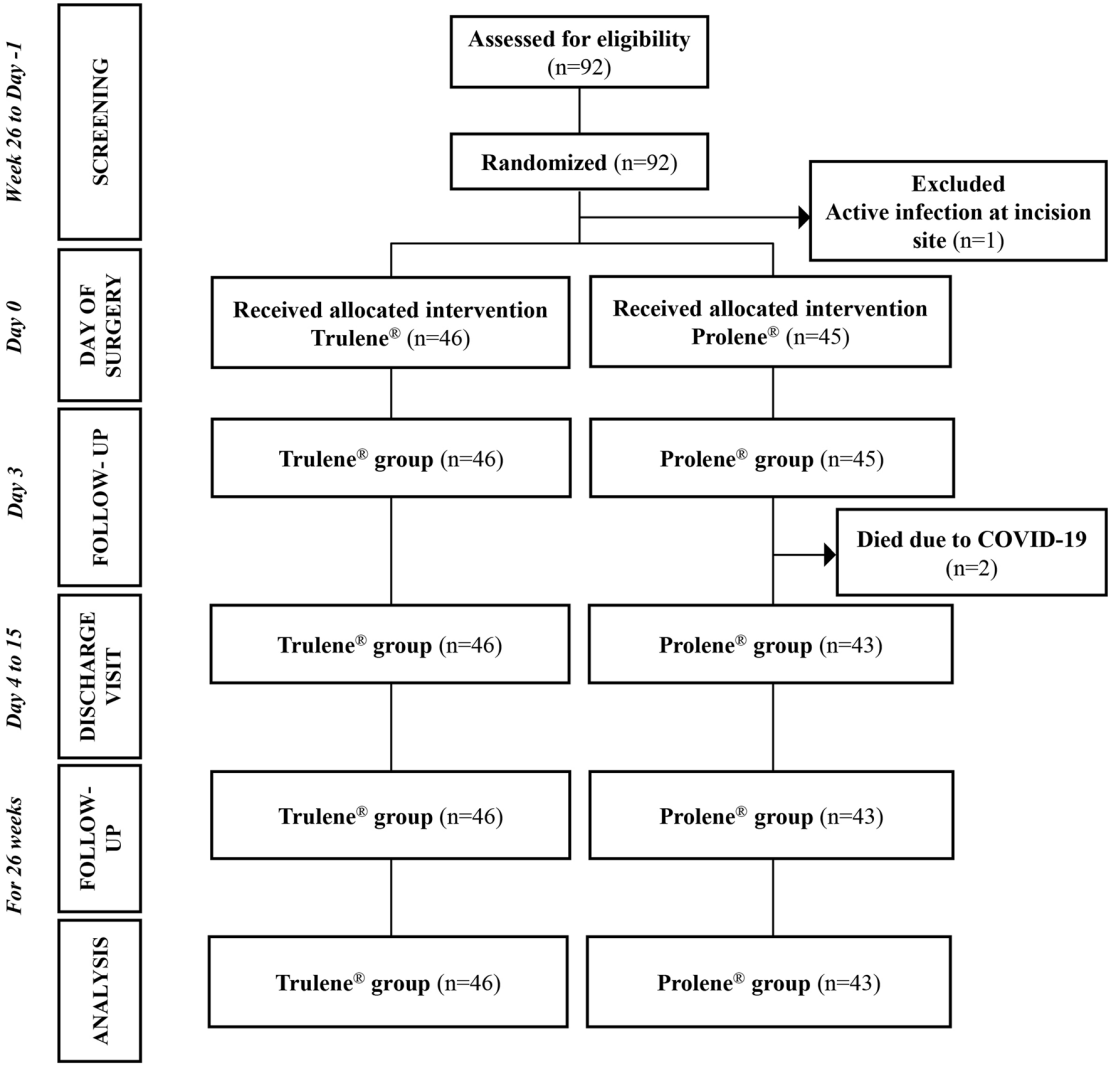
CONSORT flow chart of study participants

Between 21^st^ August 2020 and 26^th^ March 2021, 92 subjects were screened for study inclusion. The follow-up of the last subject was completed on 18^th^ September 2021. A total of 91 subjects were randomized and included in the study. One subject had active infection around the skin incision site, hence was excluded after screening. The PP analysis set consisted of 89 randomized subjects, who completed the study. The remaining 2 subjects, randomized to the Prolene® group died after Day 3 due to COVID-19 infection. All the 89 subjects received the allocated intervention (Fig. 1).

### Baseline demographics and other relevant characteristics

All the subjects participated in the study were Indians, except 2 (4.65%) subjects in Prolene® group, who were Asians (p = 0.14). A total of 80 (89.89%) males and 9 (10.11%) females were included in the study. A total of 10 (11.24%) and 11 (12.36%) subjects had a history of alcohol consumption (p = 0.58) and smoking (p = 0.40) respectively. None of the subjects that participated in the study had a history of PCI. One (2.17%) subject in the Trulene® group had a family history of cardiovascular disease (Ischemic Heart Disease) (p = 0.33). Forty (86.96%) subjects randomized to the Trulene® group and 36 (83.72%) subjects randomized to the Prolene® group had a medical/surgical history (p = 0.67). Age distribution of the subjects and ASA grading showed significant differences (p < 0.05) between the treatment groups. In both the treatment groups, more subjects were classified as ASA III. The baseline characteristics, reasons for surgery and medical/surgical history of the study participants are summarized in Table 1. The physical examination of subjects appeared normal; however, the cardiovascular system appeared to be abnormal in all the subjects.

**Table 1:** Subject demographics and other relevant characteristics among Trulene® and Prolene® groups

Subject characteristics	Trulene® (n = 46)	Prolene® (n = 43)	p value
*Demographics*			
Gender, n (%):			0.81
Male	41 (89.13)	39 (90.70)	
Female	5 (10.87)	4 (9.30)	
Age (years), mean ± SD	57.48 ± 11.90	63.75 ± 7.64	0.004*
*Anthropometrics*			
Weight (kg), Mean ± SD	69.98 ± 10.96	68.11 ± 10.18	0.34
Height (cm), Mean ± SD	164.84 ± 9.03	163.14 ± 7.71	0.41
BMI (kg/m^2^), Mean ± SD	25.83 ± 4.17	25.55 ± 3.24	0.72
*Vital signs*			
Pulse rate (beats per minute), Mean ± SD	81.74 ± 10.86	83.37 ± 12.90	0.52
Respiratory rate (respiration per minute), Mean ± SD	19.00 ± 2.68	19.49 ± 2.25	0.47
Systolic blood pressure (mmHg), Mean ± SD	122.96 ± 15.87	129.98 ± 18.36	0.06
Diastolic blood pressure (mmHg), Mean ± SD	73.91 ± 91.15	74.70 ± 10.36	0.71
*Other characteristics*			
ASA classification, n (%):			0.01*
ASA II	1 (2.17)	9 (20.93)	
ASA III	45 (97.83)	34 (79.07)	
NYHA grade, n (%):			0.56
NYHA I	1 (2.17)	0	
NYHA II	21 (45.65)	18 (41.86)	
NYHA III	24 (52.18)	25 (58.14)	
Cardiac disease and reason for surgery, n (%):			0.43
Left main disease only	0	1 (2.33)	
Left main disease in combination with a double vessel disease	0	2 (4.65)	
Left main disease in combination with a multi vessel disease	5 (10.86)	3 (6.98)	
Double vessel disease only	6 (13.05)	4 (9.30)	
Multivessel disease	35 (76.09)	33 (76.74)	
*Medical/surgical history*			
Endocrine, nutritional and metabolic diseases:			0.81
Diabetes mellitus	28 (60.87)	26 (60.47)	
Hypothyroidism	6 (13.04)	2 (4.65)	
Dyslipidemia	1 (2.17)	1 (2.33)	
Diseases of the circulatory system:			0.14
Hypertension	31 (67.39)	29 (67.44)	
IHD/CAD	13 (28.26)	13 (30.23)	
Myocardial Infarction	3 (6.52)	1 (2.33)	
Heart failure with midrange ejection fraction	1 (2.17)	0	
Triple Vessel Disease	1 (2.17)	0	
Cardiac Illness	0	1 (2.33)	
Diseases of the musculoskeletal system and connective tissue:			0.33
Left femur fracture and surgery	0	1 (2.33)	
Hip-joint replacement	0	1 (2.33)	
Diseases of the digestive system:			0.32
OesophagoGastroDuodenoscopy and *H. Pyroli* infection	1 (2.17)	0	
Diseases of the respiratory system:			0.35
Asthma	1 (2.17)	0	
Tuberculosis	0	1 (2.33)	
COVID-19	1 (2.17)	3 (6.98)	
Diseases of the nervous system:			0.60
Cerebrovascular accident	1 (2.17)	1 (2.33)	
Parkinsonism	1 (2.17)	0	
Diseases of the eye and adnexa:			0.30
S/P bilateral cataract surgery	0	1 (2.33)	
Diseases of the genitourinary system:			0.33
Benign prostatic hyperplasia	1 (2.17)	0	
Chronic kidney disease	1 (2.17)	0	

Bold numerals indicate P-values are </= 0.05

*ASA* American Society of Anaesthesiologists, *IHD/CAD* Ischemic Heart Disease/Coronary Artery Disease, *NYHA* New York Heart Association, *S/P* Status Post, *SD* standard deviation

*p < 0.05

### Primary endpoint analysis

There was no difference in the findings of primary endpoint between the groups. Incidence of myocardial infarction, stroke, renal failure and cardiac death was not recorded in any of the study participants.

#### Subgroup analyses

Heterogeneity was noted in age distribution and ASA classification between the groups. Therefore, subgroup analysis of primary endpoint with respect to these parameters was done. Subgroup analyses for subjects were similar regarding MACCE vs. age (subgroups < 60 and ≥ 60 years), and MACCE vs. ASA (subgroups ASA II and ASA III classification).

### Secondary endpoint analysis

#### Intraoperative characteristics

Left internal mammary artery was used in majority of subjects, followed by saphenous vein graft and radial artery. Left internal mammary artery was used in 43 (93.48%) subjects of the Trulene® group, and in 41 (95.34%) subjects of the Prolene® group. Saphenous vein graft was used in 34 (73.91%) and 32 (74.42%) subjects of Trulene® and Prolene® groups respectively. Radial artery was used only in 3 (6.52%) subjects randomized to Trulene® group. Table 2 summarizes the intraoperative profile parameters of the study participants. In Trulene® arm, 6-0, 7-0 and 8-0 sutures were used in a total of 45 (97.83%), 30 (65.22%), and 45 (97.83%) subjects respectively. Similarly, suture sizes 6-0, 7-0 and 8-0 were used in 43 (100.0%), 39 (90.70%), and 33 (76.74%) subjects respectively of Prolene® group (p = 0.23). Mean number of 7-0 and 8-0 sutures used were found to be significantly different (p < 0.05) between the groups (Table 2). Perioperative complications were noted in Prolene® group. One (2.33%) subject appeared to have hemodynamic instability and 1 (2.33%) subject had ventricular ectopics during the surgery (p = 0.23). The IABP was used in the subject, who had hemodynamic instability perioperatively. However, outcome of surgery for all the subjects was marked as good by the Investigators of both sites.

**Table 2:** Intraoperative and post-operative profile of the study participants

Subject profile	Trulene® (n = 46)	Prolene® (n = 43)	p value
*Intraoperative*			
Intervention approach, n (%):			0.62
Mini-thoracotomy	3 (6.52)	1 (2.33)	
Sternotomy	43 (93.48)	42 (97.67)	
Type of operation, n (%):			0.11
Off-pump CABG	46 (100.00)	40 (93.02)	
On-pump CABG	0	3 (6.98)	
Number of grafts, mean ± SD	3.04 ± 0.70	3.00 ± 0.79	0.63
Number of anastomoses, Mean ± SD	4.98 ± 1.36	4.91 ± 1.44	0.77
Number of blood transfusions, Mean ± SD	0.22 ± 1.47	0.07 ± 0.34	0.52
Total operative time (hours), mean ± SD	5.45 ± 1.58	5.13 ± 1.45	0.33
Number of 6-0 sutures used, mean ± SD	2.76 ± 1.86	2.51 ± 1.35	0.49
Number of 7-0 sutures used, mean ± SD	1.93 ± 0.52	2.79 ± 1.34	0.001*
Number of 8-0 sutures used, mean ± SD	3.13 ± 2.13	1.70 ± 1.05	0.001*
*Post-operative*			
ICU stay (days), Mean ± SD	5.26 ± 1.89	5.65 ± 1.62	0.74
Hospital stay (days), Mean ± SD	6.41 ± 1.47	6.21 ± 1.39	0.71
Readmission, n (%)	1 (2.17)	1 (2.33)	0.33

Bold numerals indicate P-values are </= 0.05

*CABG* coronary artery bypass graft surgery, *ICU* intensive care unit, *SD* standard deviation, *p < 0.05

**Fig. 2:**
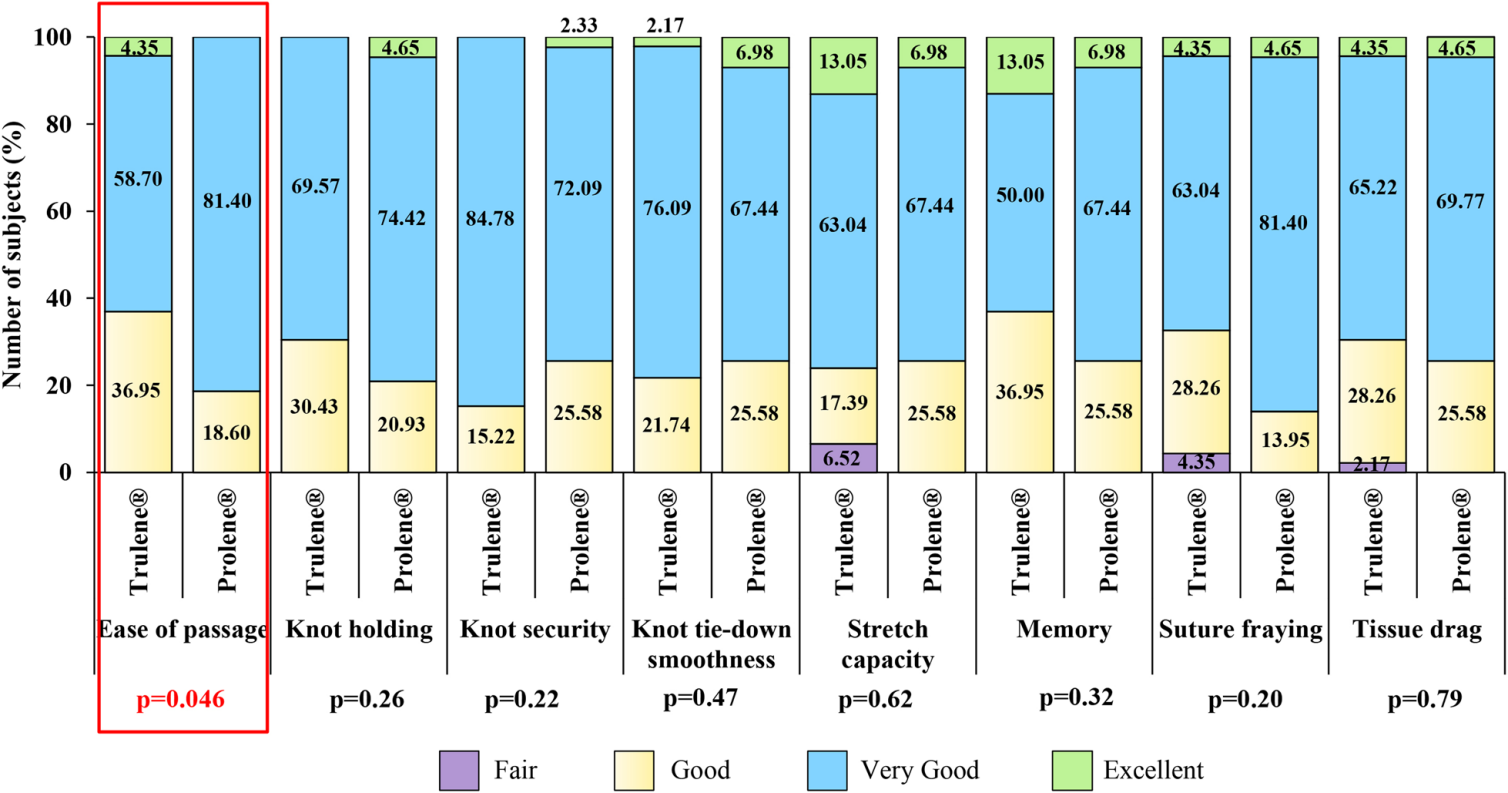
Intraoperative suture handling parameters of the subjects randomized to Trulene® (n = 46) and Prolene® (n = 43) groups: p < 0.05 is statistically significant

The results relative to intraoperative suture handling characteristics are shown in Fig. 2. A significantly lower (p < 0.05) “Excellent” and “Good” score for ease of passage was registered during surgery in the subjects of Prolene® group, compared to the Trulene® group. The values of the other seven characteristics of intraoperative suture handling of the subjects obtained for each suture group were comparable. In addition, perioperative suture-related challenges were noted in both treatment arms. The Investigator showed dissatisfaction, due to broken suture and needle came off the suture in 2 (4.35%) subjects of Trulene® group, while due to broken suture (8-0 suture) and small needle (7-0 suture) in 1 (2.33%) subject of Prolene® group.

#### Post-operative characteristics

By week 6 post-DOD, 1 (2.17%) subject of Trulene® group reported superficial SSI. The subject was readmitted to hospital, and the incidence was reported as serious adverse event. One (2.33%) subject in the Prolene® group was also readmitted to the hospital due to COVID-19 infection during week 6 post-DOD. No incidence of deep SSI was noted in both the groups. After the surgery, reintervention, anastomotic revision, revascularization, and repeated procedure were not required in any subject of both groups. The median time taken to return to work was 14 days in both Trulene® and Prolene® groups, while median time taken to return to normal day to day activities was 14 days in Trulene® group, and 12 days in Prolene® group. Other post-operative characteristics of the subjects are presented in Table 2.

Details of some of the analgesics, antiplatelet medications and antibiotics, which were prescribed to most of the subjects during the study period is shown in Table 3.

**Table 3:** Concomitant or prescribed medication details in study participants during the study period

Prescribed medications	Trulene® (n = 46)	Prolene® (n = 43)
*Analgesics/antiplatelet, n (%)*		
Aspirin	45 (97.83)	43 (100.00)
Clopidogrel	36 (78.26)	29 (67.44)
Paracetamol	32 (69.56)	31 (72.09)
Tapentedol	17 (36.96)	15 (34.88)
Aspirin + Clopidogrel	11 (23.91)	13 (30.23)
*Antibiotics, n (%)*		
Piperacillin + Tazobactam	33 (71.74)	33 (76.74)
Levofloxacin	31 (67.39)	36 (83.72)
Cefixime	20 (43.48)	16 (37.21)
Cefuroxime	12 (26.09)	13 (30.23)

#### Subject satisfaction score and QoL

**Fig. 3:**
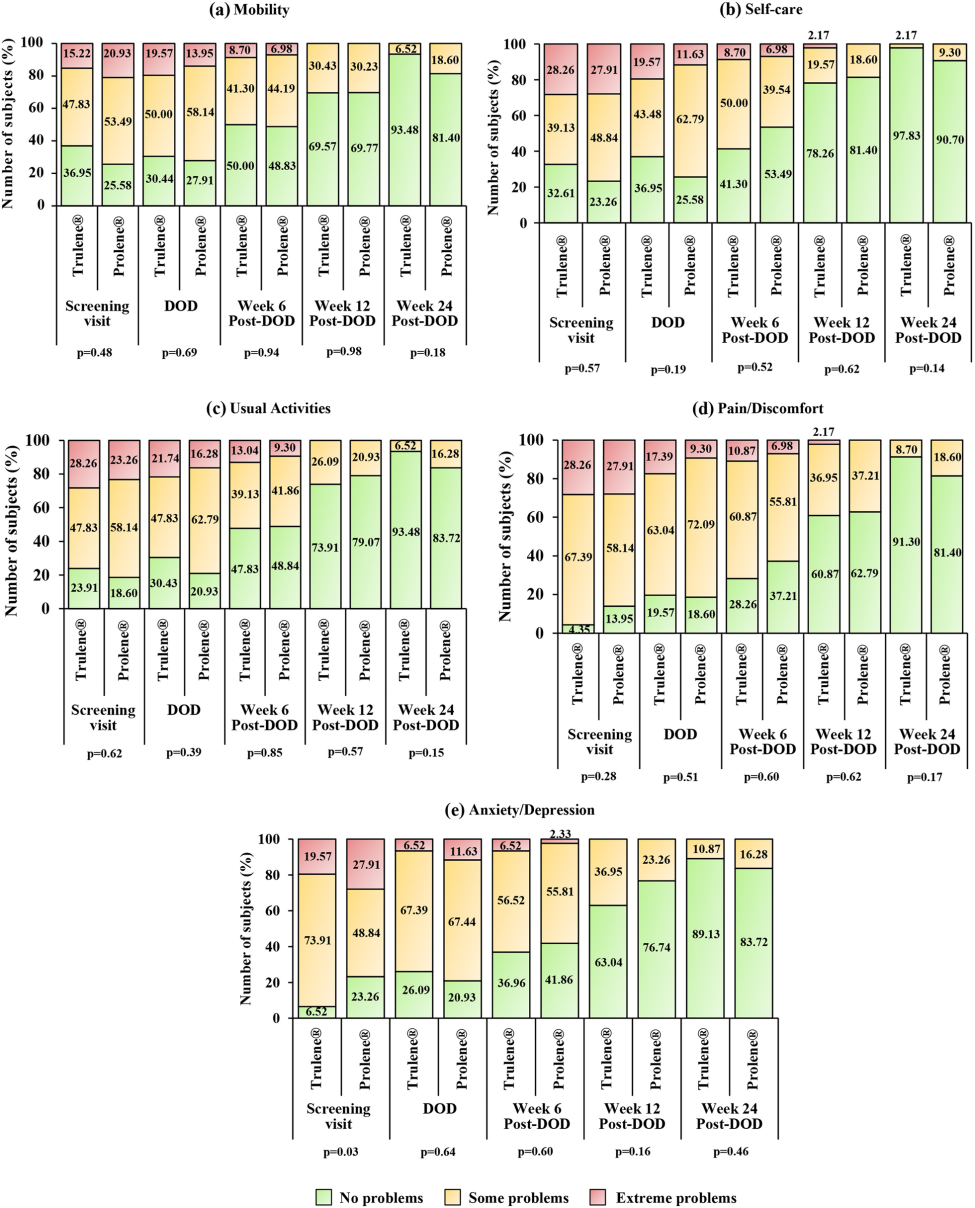
Collated responses to EQ-5D 3L in subjects randomized to Trulene® (n = 46) and Prolene® (n = 43) groups: **a** Question 1: Mobility, **b** Question 2: Self-care **c** Question 3: Usual activities **d** Question 4: Pain/discomfort **e** Question 5: Depression/anxiety. *DOD* day of discharge. p < 0.05 is statistically significant

In all five items of the EQ-5D 3L, QoL was found to be impaired at the screening visit and then gradually improved through every post-operative visit (Fig. 3). At screening visit, the number of subjects with no problems (level 1), some problems (level 2), and extreme problems (level 3) in terms of mobility, self-care, usual activities, and pain/discomfort were comparable between the treatment groups. However, a significant difference (p < 0.05) in anxiety/depression was recorded between the subjects of Trulene® and Prolene® groups. A lower proportion of subjects with moderate problems were observed in both the groups during every follow-up visit post-surgery compared to the screening visit. Apparently, the proportion of subjects with no problems for each QoL dimension increased with every post-surgery follow-up visit in comparison to the screening visit.

**Fig. 4:**
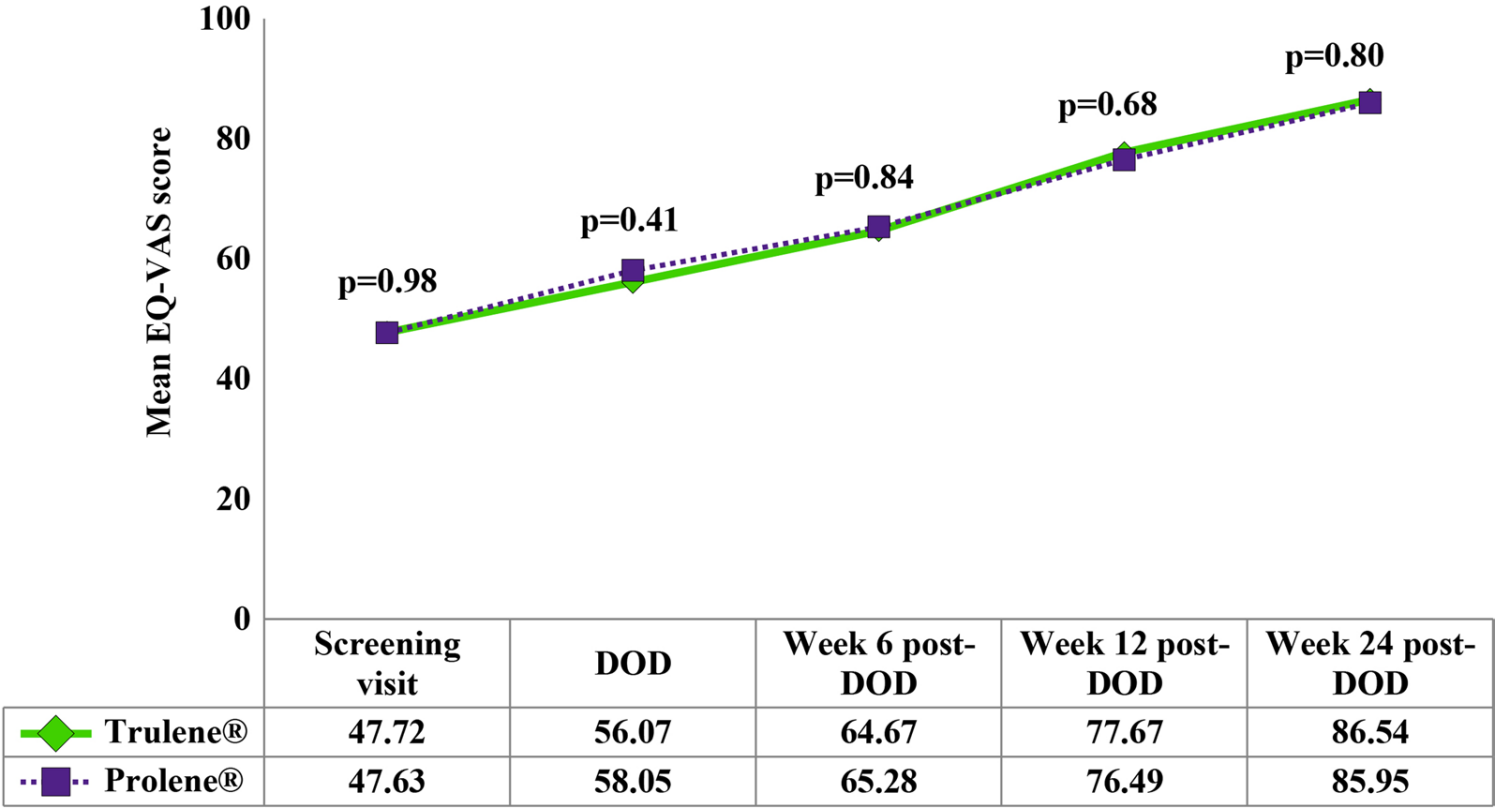
Changes in EQ-VAS across follow-up visits in subject of Trulene® (n = 46) and Prolene® (n = 43) groups. *DOD* day of discharge

EQ-VAS score is considered to derive information about the respondents’ subjective health perception. The measured EQ-VAS score was similar in both groups at every visit. The mean values of the EQ-VAS score were gradually improved with each post-surgery follow-up visit (Fig. 4).

#### Adverse events and serious adverse events

A total of 11 (12.36%) adverse events (not-related to device) and 2 (2.25%) serious adverse events occurred in a total of 89 subjects. In Trulene® group 2 (4.35%) cold, 1 (2.17%) cough and vomiting, 1 (2.17%) upper respiratory tract infection and 1 (2.17%) serious adverse event (hospitalization due to SSI) was recorded. Similarly, in Prolene® group 3 (6.98%) fever, 1 (2.33%) ventricular ectopic beats, 1 (2.33%) hemodynamic instability, 1 (2.33%) fatigue, 1 (2.33%) general body pain and 1 (2.33%) serious adverse event (hospitalization due to COVID-19 infection) was recorded. Both serious adverse events occurred at week 6 post-DOD and the event was not attributed to the medical device, and both the subjects recovered within the study period, and continued the study. None of the subjects had adverse device effects, serious adverse device effects, or unanticipated serious adverse device effects.

## Discussion

In this study, although, both the groups were comparable with respect to demographics and vital signs, significant difference in age distribution and ASA classification may have occurred due to randomization of subjects with large variability in the age ranges (> 18 years), requiring elective primary CABG surgery. However, the heterogeneity has not impacted the results of primary endpoint of this study, as mandated by subgroup analysis. The hypothesis of the present study was that if incidence of MACCE after using Trulene® suture is lower or equal to the incidence of MACCE after using reference suture (Prolene®), the suture material Trulene® can be regarded as a safe and efficient alternative to polypropylene suture Prolene® used for CABG surgery. Indeed, the result indicated clinical equivalence of Trulene® suture, as non-significant difference was found in the primary and secondary endpoints of the study between the two groups.

One of the world's leading causes of mortality, morbidity, and disability is cardiovascular disease, attributing to 32% of all global deaths [21, 22]. As per the Global Burden of Disease study state age-standardized death rate of cardiovascular disease was reported to be 272/100,000 people in India, which is higher than the global average of 235 [23]. CABG surgery is one of the safest and most commonly performed elective surgical procedures, and provides steady improvements in survival; but it also causes post-operative complications, such as death, myocardial infarction, stroke, wound infection, prolonged requirement for mechanical ventilation, acute kidney injury, and bleeding [24], affecting patient’s QoL, and leading to morbidity and mortality [4]. Myocardial infarction, stroke, death along with repeat revascularization is defined as MACCE. A previous study (SYNTAX trial) compared CABG surgery with PCI in 1800 patients, who had multi-vessel disease or left main disease. The study observed the following 5 years complication rates for CABG: myocardial infarction (3.8%), death (11.4%), stroke (3.7%), revascularization (13.7%), and MACCE (26.9%); and for CABG vs. PCI: myocardial infarction (9.7%), death (13.9%), stroke (2.4%), revascularization (25.9%), and MACCE (37.3%) [2]. A study involving Optilene® suture (consisting of polypropylene 95% and polyethylene 5%) showed occurrence of six CABG events: one death (hemorrhagic shock), two myocardial infarctions and three renal failures, leading to a CABG event rate of 3% [2]. In this study there was no incidence of MACCE at any point of follow-up among the two groups. A previous study reported use of both off-pump and on-pump surgery in patients of Germany, Spain and Italy, who underwent elective CABG surgery for revascularization. But, a centre-specific effect was observed regarding off-pump surgery in Germany, where 91% of the interventions were performed as off-pump surgery, compared to 33% in Italy and 41% in Spain [2]. A likely higher number of off-pump CABG surgeries were performed in subjects of the present study. In addition, sternotomy was used as the intervention approach in majority of subjects undergoing CABG surgery. Left internal mammary artery graft followed by saphenous vein graft was used in maximum number of study participants of Trulene® as well as Prolene® group. Comparable results for handling of the suture thread were found, regarding, knot holding, knot security, knot tie-down, stretch capacity, memory, suture fraying and tissue drag. However, a significant (p < 0.05) difference in ease of passage was noted between the groups. Ease of handling of the suture material was rated as “Excellent” in majority of subjects of Trulene® group, whereas, more “Very good” scores were found in Prolene® group. In this study, a typical population with cardiac disease was treated. Anastomotic revision, reintervention, revascularization and repeated procedure were not required during the study period, and good outcome of surgery was registered for both the treatment arms at the end of the surgery.

A recent review demonstrated the common reason for readmissions after 30 days of CABG were infection and sepsis (6.9–28.6%), cardiac arrhythmia (4.5–26.7%), congestive heart failure (5.8–15.7%), respiratory complications (1–20%) and pleural effusion (0.4–22.5%) [25]. A study done by Jadhao et al. compared incidence of sternal wound SSIs among stapler and polyamide suture groups, and found higher incidence of SSIs in stapler group (9.72%) than the polyamide suture group (7.17%) [26]. In the present study, incidence of superficial SSI was recorded by 6^th^ week post-DOD in one subject of the Trulene® group. However, the SSI was not associated with the medical device, and the subject recovered by the next visit.

Clinically, it is necessary to follow the subjects regarding care processes, discharge, post-discharge care coordination, recovery and ability to return to everyday life. In the present study, the length of intensive care unit was comparable between the treatment groups. A maximum of 10 days of hospital stay was noted in both groups. Furthermore, the time taken to return to normal day to day activities, and return to work was comparable between both the suture groups. In addition, QoL of the subjects improved from preoperatively to 26 weeks post-operatively. This increase was independent of the type of surgery (off-pump or on-pump), and type of chest opening (sternotomy or mini-thoracotomy). Regarding the safety, the type of adverse events in both arms was of low risk, and not related to the suture material. Although, serious adverse events were observed in the study, but those were not considered to be related to the study device.

This study has some limitations; firstly, the surgeons were not blinded, which might have led to favoring one suture or another while assessing the intraoperative suture handling. Secondly, the CABG procedures are clean surgeries, and the risk of SSI is generally minimal. Infection can only originate from contaminants in the operation room or from the surgical team, or most commonly from skin colonists. Multiple testing measures were used in this study without adjustment of p-value, which might increase the probability of type I error. However, the study is methodologically robust to detect a difference for the primary and secondary outcomes, which indicates that the use of Trulene® suture can be generalized and validated to a wider population. The study outcomes further indicated that Trulene® suture can be used in all surgeries indicated for Prolene® suture.

## Conclusion

The results of primary and secondary endpoints indicate that the Trulene® and Prolene® sutures are clinically equivalent, as non-significant differences were seen in cumulative incidence of MAACE, anastomotic revision, incidence of SSI, total operative time, length of post-operative hospital stay, revascularization, subject satisfaction score and QoL, return to work and normal day to day activities, intraoperative suture handling parameters (except ease of passage), and adverse events among the groups. Both Trulene® and Prolene® polypropylene sutures are safe and effective for anastomosis construction in CABG surgery using a routine clinical procedure.

## Data Availability

All data generated or analyzed, and the materials used during the study are available from the corresponding author AG on reasonable request.

## Abbreviations

CABG: Coronary artery bypass graft surgery
CAD: Coronary artery disease
CONSORT: Consolidated Standards of Reporting Trials
CTRI: Clinical Trial Registry of India
EQ-VAS: EuroQoL-visual analogue scales
DOD: Day of discharge
IABP: Intra-aortic balloon pump
ICH-GCP: International Conference on Harmonization of Technical Requirements-Good Clinical Practice
MACCE: Myocardial infarction, stroke, renal failure and cardiac death
PCI: Percutaneous coronary intervention
SD: Standard deviation
SSI: Surgical site infection
QoL: Quality of life
